# The Next Step to Further Decrease Veterinary Antibiotic Applications: Phytogenic Alternatives and Effective Monitoring; the Dutch Approach

**DOI:** 10.3389/fvets.2021.709750

**Published:** 2021-09-03

**Authors:** Maria J. Groot, Bjorn J. A. Berendsen, Natalie B. Cleton

**Affiliations:** Wageningen Food Safety Research, Wageningen University & Research, Wageningen, Netherlands

**Keywords:** phytogenics, antibiotics, monitoring, Netherlands, strategy

## Abstract

Antibiotics are used to control infectious diseases in both animals and humans. They can be life-saving compounds but excessive use in animal husbandry leads to the development of antibiotic resistance which can impact the public health. Since similar antibiotics are used in both animal and human healthcare, it is important to reduce the use of antibiotics in production animals. In the Netherlands policies have been developed aiming for a decrease of antibiotic usage in animals, and alternatives to antibiotics are investigated. Currently, a one-on-one relationship between farmer and veterinarian is successfully implemented and (national) registration of antibiotic usage is mandatory. Unfortunately, after a 70% decrease in antibiotic usage since 2009, this decrease is now stagnating in most sectors. Innovative strategies are required to facilitate a further reduction. One promising option is a focus on farm management and natural alternatives to antibiotics. The Dutch government has invested in the spread of knowledge of natural remedies and good animal management to support animal health via so called Barnbooks for farmers and veterinarians. Another option is the analysis of on-farm antibiotic use to prevent unregistered applications. New (bio)analytical strategies to monitor the correct and complete registration of antibiotic usage have been developed and trial-tested in the Netherlands. Such strategies support a risk-based monitoring and allow effective selection of high-risk (high antibiotic use or illegal antibiotic) users. Both effective monitoring and the availability and knowledge of alternatives is a prerequisite to achieve a further significant decrease in antibiotic veterinary usage.

## Introduction

In the Netherlands, strategies have been developed aiming to decrease use of antibiotics in animals. In response to several outbreaks of multi-resistant bacteria and the resulting societal and political pressure, several compulsory and voluntary measures were initiated to reduce antibiotic usage by farmers and prescription by veterinarians (1, 2). These measures have led to a decrease of antibiotic use of almost 70% in the past 10 years (3). Further decrease might be achieved by optimizing farm management and housing, the application of natural products to improve animals health, and effective monitoring of antibiotic use and registration. In this overview we discuss the use of plant based products which have become part of farm health plans in the Netherlands and monitoring of antibiotic use to detect non-registered applications.

## Dutch Reduction of Antimicrobials by Almost 70% Now Stagnant in Most Sectors

Antibiotics are used to control infectious diseases in both animals and humans. They can be life-saving compounds but excessive use in animal husbandry contributes to the development of antibiotic resistance which impacts public health. Since similar antibiotics are used in both animal and human healthcare, it is important to reduce the use of antibiotics in production animals (4). In recent decades, these risks to public health have been seen with the identification of livestock associated methicillin-resistant *Staphylococcus aureus* and extended spectrum beta-lactamase-producing bacteria (5, 6). “Therapeutic antimicrobial use in farm animals in the Netherlands doubled between 1990 and 2007” (1). Next to the development of antibiotic resistance, excessive use of antibiotics in livestock may lead to residues in animal products impairing human health. These and other events have contributed to more pressure from the public to increase the monitoring and control of the use of antimicrobials in Dutch farms.

“The Netherlands Veterinary Medicines Authority” (SDa), an independent body, was established in 2010 with the main aim of reducing antimicrobial use in the farm industry (3). The SDa annually reports the use of antimicrobials in farm animals in the Netherlands. The SDa is a partnership between the government, the Royal Dutch Veterinary Association (KNMvD) and livestock industries, with the aim to monitor antibiotic usage on farms and set reduction targets. To achieve this, multiple goals were set comprising of monitoring current antimicrobial use on individual farms and at veterinarian level and creating benchmark indicators to identify high risk users and prescribers. The body was granted the authority to apply disciplinary sanctions and extra monitoring of high users and prescribers by the Dutch Food and Consumer Product Safety Authority (NVWA).

Additional legislation was set in 2014 which prescribed that only veterinarians were allowed to administer antimicrobials. Exceptions are possible under strictly regulated and monitored conditions. The goal was to create closer interaction between veterinarian and farmer, compulsory herd health checks and the presence of a farm health plan and farm therapeutic plan. Currently, a one-on-one relationship between farmer and veterinarian has been successfully implemented and (national) registration of antibiotic usage is mandatory.

**Figure 1:**
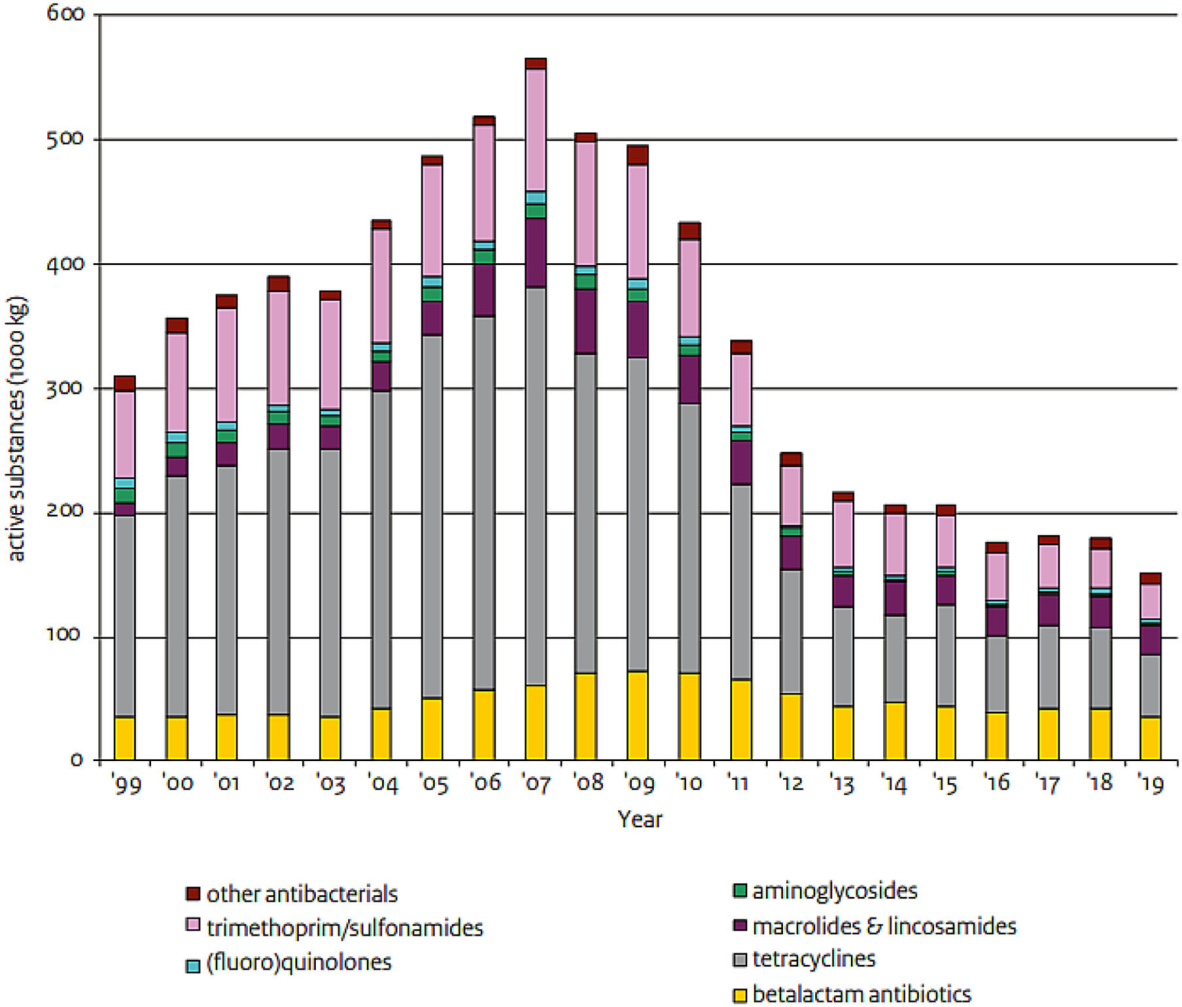
Antimicrobial veterinary medicinal product (AVMP) sales 1999–2019 in kg (thousands). Trends in antibiotic sales from 1999 to 2019 in The Netherlands. Reproduced from NethMap (7).

The current figures (Figure 1) show that the Dutch approach to antimicrobial use reduction set up by the government, farmers and veterinarians has resulted in a reduction of antimicrobials by almost 70% in 2019 compared to 2009. While in some sectors (veal calves) this decrease is still occurring, in other sectors (dairy cattle, broilers, and swine) this decrease is now slowing down or stagnating (3). Further reduction in antimicrobial use will require an animal sector-specific approach as there are different trends in the various sectors. Some sectors (like broiler and pig farms) show many farmers in the low usage category and a small trail of high users. Other sectors (like veal farms) show a generalized high use of antimicrobials, which would therefore justify a more general approach for that whole sector. Strategies to further reduce use of antimicrobials will therefore depend on the sector and will require a broader approach to farm animal health. This will include looking at alternatives to antimicrobials but also housing and ventilation methods, focusing on preventative strategies to yield a further decrease. Also, non-registered use should be prevented and therefore an effective monitoring system is indispensable.

## Use of Phytogenics to Keep Animals Healthy

Phytotherapeutics are plant-based medicine that were traditionally used to prevent and cure disease in humans and livestock (8–10). Nowadays in animal husbandry phytotherapeutics are mostly not registered as veterinary medicine but used as complementary feed or feed additives. Since the word phytotherapeutics may lead to the suggestion that these products are veterinary medicine, phytogenics (plant derived) products is more appropriate.

Phytogenics can be a huge resource for improving animal health since bioactive plant components can work on gut flora, modulate the immune system, affect digestion and many other systems, exerting a multi-target action (11–13). The control of multi-drug resistance in human and animal pathogens might profit from the multi-target action of plant-based products (14).

The Dutch government invested in providing information for farmers and veterinarians concerning alternatives in management and use of health supporting products. Medicinal plants have been used worldwide for prevention and treatment of diseases in animals and humans for ages. The broad spectrum of plant metabolites represents a huge potential for medication of herbivore and omnivore livestock. While there is a large amount of evidence-based knowledge about medicinal plants published in literature in both English and German language (8), this is hard to access for Dutch farmers.

To make the information on alternatives and their possible application accessible for a wider public, so-called “Barn books” per animal category were published by WFSR in a project funded by the Dutch Ministry of Agriculture, Nature and Food Quality. First books were aimed at organic farmers and focussed on dairy cattle, poultry and pigs. These books are also translated into English as Natural Dairy Cow Health, Natural Swine health, Natural Poultry Health, Guide to keeping your animals healthy with herbs, and other natural products (15–17). These books are freely available via the internet. Since conventional farmers also needed to reduce the use of antibiotics, the series was extended in a governmental project and consists at the moment of books for dairy cows, swine, poultry, turkeys, rabbits, veal calves, dairy goats and sheep (18–25). The aim was to provide both farmers and their veterinarians with objective information on products and what to expect from their effects.

The books consist of chapters per life stage of the animal with general management advice and natural products that can support the animals' health or prevent the animals from becoming sick. The products described are complementary feeds or feed additives. In the annexes there is a list of herbs with their main constituents and use, and per product general information and information from research with references is provided. The barn books give information on products for which the supplier provided the full composition of the product and results from field trials and literature. Products with no full disclosure of the composition or without underlying substantiation are not included. The aim was to provide both farmers and their veterinarians with objective information on products and their expected effects.

Natural products used as feed additives have shown to improve zootechnical parameters such as growth performance, feed conversion and gut health in livestock, and so reduce the need for antibiotics (26–31). The use of natural products such as phytogenics and other natural products alone cannot provide the solution for the use of antibiotics in animal husbandry. In animal production most antibiotics are used in young animals (broilers, pigs, turkeys, and veal calves) for disorders of the intestinal or respiratory tract. The immune system of young animals is not yet fully developed in the first weeks of live and in combination with insufficient intake of colostrum, contact to pathogens often gives rise to high morbidity and mortality. Inadequate management, such as long distance transports, fasting, mingling animals from different sources, high stock density, changes in climate, inappropriate diets, and suboptimal hygiene play a role in the susceptibility to disease of these animals. Therefore, other principles that can be used to reduce the need for antibiotics are breeding (slower growing breeds, more resilient animals, optimal instead of maximum production, dual purpose breeds), management (hygiene, feeding, housing, climate, stock density), quality control and chain management and a quality based payment system (low residue animals get a bonus), and development of antibiotic free production chains. In some aspects the abovementioned principles are already used in organic farming, where due to European regulations the use of antibiotics is limited, production is in most cases lower than in conventional farms, stock density is lower and organic products get a better price. Because the use of antibiotics in organic production is limited by European regulations on organic management, natural healing methods like phytotherapeutics, vitamins, and minerals are used to keep the animals healthy or treat when sick.

## Analytical Strategies That Support and Stimulate Prudent Use of Antibiotics

Next to management and phytogenics, (on-site) analysis of antibiotic residues to check for correct registration of antibiotic usage will prevent unregistered application and thus contribute to the prudent use of antibiotics.

Compulsory registration of antibiotic use is an important step toward monitoring antibiotic use on farm level (1). However, such a measure can only achieve its full potential and effectiveness if registration of antibiotic use is enforced. An important tool that can be applied is the analysis of antibiotic residues in non-invasive matrices (matrices that can be taken from live animals) such as hair and feathers (32, 33). We expect that this tool will become even more effective if analysis is done on-site so that appropriate actions can be taken immediately. This measure will also have a preventive effect and contribute to the prevention of unregistered use of antibiotics.

Routine monitoring of veterinary drug residues is most commonly carried out in the slaughter phase. Animal tissue is sampled, transported to the laboratory and analyzed for violation of the maximum residue limit. Sampling is also done at the farm. Usually urine, milk or eggs depending on animal type and production use are collected. Taking urine samples can be quite time-consuming. Excretion studies have shown that many antibiotics have a relative high excretion rate in urine and that residues can only be detected shortly after administration. Therefore, the analysis of urine samples to check legally correct antibiotic use, is not an economically feasible and practical strategy.

Studies have demonstrated that antibiotic residues are present in hairs and feathers in relatively high concentrations (34). As residue excretion from feathers is slow (32, 34–37) and predominantly occurs during molting, feathers make an ideal matrix for long term monitoring. Studies in broilers demonstrated that a decrease in the concentration of residues in feathers after dispositioning (after administration of antibiotics) is mainly the result of the growth of the animal and the increase of feather weight, rather than excretion of the drugs from the feathers. We previously demonstrated that in practice, feather analysis is an effective strategy to determine the antibiotics that broilers were exposed to throughout their (short) lives (33). This strategy also applies to hairs of calves and swine. Due to these animals having a much longer life compared to broilers, most likely not all antibiotics administered throughout the whole lifetime of the animal can be detected at the slaughter age. Note, that for some antibiotics, residues were even detectable up to 100 days after administration in hair.

As hair and feather samples are rather easy to collect on site and contain as well as retain information about the history of antibiotic use in the animal (33), these samples are ideal for antibiotic use detection to check for correct use and registration. In a pilot study that included 20 broiler farms, two cases of antibiotics residues were detected in feathers for which no recent applications were registered. This pilot demonstrated the potential of using feathers for antibiotic residue detection.

Additional research (not published) demonstrated that, for screening of antibiotics, cotton pads could also be used to obtain samples from calves. Instead of shaving hair (which is an elaborate procedure), these cotton pads were wiped over the animal's neck or back and stored in a tube. The (qualitative) analytical results of the cotton pad samples proved to be comparable to the results of the hair samples taken from the same animals. This demonstrated that also a quick and easy sample taking procedure can be applied for cattle and swine.

Even though the above presented approach has been proven successful in finding non-registered use of antibiotics, a main disadvantage of this approach is that the whole procedure (sampling, transport, and analysis) is time consuming and relatively expensive. We are therefore, currently investigating the application of on-site lateral flow devices (LFD) for easy and quick analysis of antibiotics in hair, feathers and preferably cotton pad swipes.

LFDs for the detection of antibiotics are commercially available (e.g., CHARM sciences inc (Lawrence, MA, USA) and PerkinElmer (Waltham, MA, USA), and can also be easily prepared and adapted in-house, as long as residue specific binding molecules (e.g., antibodies) are available. Samples can easily be extracted on-site with a buffer and a small volume of the extract is then transferred onto a LFD. After several minutes, a qualitative result is obtained. LFDs can detect a single specific compound or compound class, but multiplex devices are available. This development allows inspectors (after a short training) to carry out a quick assessment of antibiotic exposure on site. After screening multiple animals, results of the LFDs are compared with the farms antibiotic use registration system and within 15 min the inspector can obtain a good indication of the status of the farm with regard to antibiotic use and registration thereof. In case no discrepancies are observed, the inspector can quickly move on to the next farm. If differences are observed, the inspector can discuss this with the farmer and, if deemed necessary take additional samples for laboratory analysis. This approach is very promising and, as it is a very quick and easy approach, is expected to have a preventive effect of misuse of antibiotics in animal husbandry. The large scale implementation to LFD application on hairs and feathers at farm level will probably present new challenges. These especially regard the application of the test in a potentially contaminated environment and the legal status of the result. The latter includes the validation of the LFD under field conditions. Those hurdles are expected to be overcome in the coming years. Nonetheless, the currently proposed strategy can easily be applied at laboratory level already.

## Conclusion

In conclusion, in the Netherlands an important decrease of antibiotic use in animal husbandry has been achieved since 2009. However, it is currently plateauing even though a further reduction is urgent. New approaches are suggested to further decrease antibiotic usage. The differences between antimicrobial use reduction achieved per sector suggest that approaches should probably be sector and sometimes farm specific. These could include optimized management and raising awareness for the risk of antimicrobial resistance. Additionally, we advocate the public availability of knowledge on natural products as alternatives and an effective strategy to monitor and enforce correct use and registration of antibiotic applications. These are promising tools to achieve lower antibiotic inputs and additional monitoring of antibiotic use data must demonstrate their effectiveness.

## Data Availability Statement

Publicly available datasets were analyzed in this study. This data can be found at: data available via the references.

## Author Contributions

MG is the responsible author and wrote the part: use of phytogenics to keep animals healthy. NC wrote the part about the Dutch policy: Dutch reduction of antimicrobials by almost 70% now stagnant in most sectors. BB wrote the part: analytical strategies that support and stimulate prudent use of antibiotics. All authors have contributed the whole article.

## Conflict of Interest

The authors declare that the research was conducted in the absence of any commercial or financial relationships that could be construed as a potential conflict of interest.

## Publisher's Note

All claims expressed in this article are solely those of the authors and do not necessarily represent those of their affiliated organizations, or those of the publisher, the editors and the reviewers. Any product that may be evaluated in this article, or claim that may be made by its manufacturer, is not guaranteed or endorsed by the publisher.
